# Observations on Luminous Animals

**Published:** 1811-05

**Authors:** J. Macartney


### *Observations on Luminous Animals.* By J. MACARTNEY, Esq.

(Phil. Trans. Part II. 1810.)

THE property which certain animals possess of emitting light is so curious and interesting, that it has attracted the attention of naturalists in all ages. It was particularly noticed by Aristotle and Pliny amongst the ancients, and the publications of the different learned societies in Europe contain numerous memoirs upon the subject. Notwithstanding the degree of regard bestowed upon the history of luminous animals, it is still very imperfect; the power of producing light appears to have been attributed to several creatures which do not possess it; some species which enjoy it in an eminent degree, have been imperfectly described or entirely unobserved; the organs which afford the light in certain animals have not been examined by dissection; and lastly, the explanations that have been given of the phænomena of animal light, are unsatisfactory, and in some instances palpably erroneous.

As this subject forms an interesting part of the history of organized beings, I have for some years availed myself of such opportunities as occurred for its investigation. Having communicated the result of some of my researches to the Right Honourable Sir Joseph Banks, he immediately offered me his assistance with that liberality, which so eminently distinguishes him as a real lover of science. I am indebted to him for an inspection of the valuable journal he kept during his voyage with Captain Cook; for permission to copy the original drawings in his possession, of those luminous animals discovered in both the voyages of Cook; and for some notes upon the luminous appearance of the sea, that were presented to him by Captain Horsburg, whose accuracy of observation is already known to this learned Society.

In the following paper, I shall first examine the grounds on which the property of shewing light has been ascribed to certain animals, that either do not possess it, or in which its existence is questionable. I shall next give an account of some luminous species, of which some have been inaccurately described, and others quite unknown. I shall endeavour to explain from my own observations, and the information communicated to me by others, many of the circumstances attending the luminous appearance of the sea. I shall then

 describe describe the organs employed for the production of light in certain species; and lastly, I shall review the opinions which have been entertained respecting the nature and origin of animal light, and relate the experiments I have made for the purpose of elucidating this part of the subject.

The property of emitting light has been reported to belong to several fishes, more particularly the mackarel, the moon-fish (tetraodon mola,) the dorado, mullet, sprat, &c.

Mr. Bajon observed during the migration of the dorados, &c. that their bodies were covered with luminous points. These however proved upon examination to be minute spherical particles that adhered to the surface of these fishes; and, he adds, appeared to be precisely the same sort of points that illuminated the whole of the sea at the time. They were therefore in all probability the minute kind of medusa, which I shall have occasion to describe hereafter.

Godeheu de Riville states, in a paper sent to the Academy of Sciences at Paris, that on opening the scomber pelamis while alive, he found in different parts of its body an oil which gave out much light: but it should be observed, that Riville had a particular theory to support, for which this fact was very convenient, and that other parts of his memoir bear marks of inaccuracy. It may be added, that if the oil of fishes were usually luminous, which Riville supposed, it would be almost universally known, instead of resting on a solitary observation.

As far as I am able to determine from what I have seen, the faculty of exhibiting light during life does not belong to the class of fishes. It appears probable, that some fishes may have acquired the character of being luminous, from evolving light soon after death.

Some species of lepas, murex, and chama, and some star-fish have been said to possess the power of shining; and the assertion has been repeated by one writer after another, but without quoting any authority.

Brugueire upon one occasion saw, as he supposed, common earth worms in a luminous state; all the hedges were filled with them; he remarked that the light resided principally in the posterior part of the body.*

Flaugergues pretended to have seen earth worms luminous in three instances; it was at each time in October; the body shone at every part, but most brilliantly at the genital organs.†

* Journal d'Histoire Naturelle, Tom. II.

† Journal de Physique, Tom. XVI.

Notwithstanding

Notwithstanding this concurrence of testimony, it is next to impossible, that animals so frequently before our eyes as the common earth worm, should be endowed with so remarkable a property, without every person having observed it. If they only enjoyed it during the season for copulation, still it could not have escaped notice, as these creatures are usually found joined together in the most frequented paths, and in the garden walks.

In different systems of natural history, the property of shining is attributed to the cancer pulex. The authorities for this opinion are Hablitzl, and Thules and Bernard. The former observed upon one occasion, a cable that was drawn up from the sea exhibit light, which upon closer inspection was perceived to be covered by these insects.* Thules and Bernard reported that they met with [illegible] number of this species of cancer on the borders of a river, [illegible]ely luminous.† I am nevertheless disposed to question the luminous property of the cancer pulex, as I have often had the animal in my possession, and never perceived it to emit any light.

The account given by Linneus of the scolopendra phosphorea, is so improbable and inconsistent, that one might be led to doubt this insect's existence, particularly as it does not appear to have been ever seen, except by Ekeberg, the Captain of an East Indiaman, from whom Linneus learnt its history.

I now proceed to the description of those luminous animals that have been discovered by the Right Honourable Sir Joseph Banks, Captain Horsburg, and myself.

On the passage from Madeira to Rio de Janeiro, the sea was observed by Sir Joseph Banks to be unusually luminous, flashing in many parts like lightning. He directed some of the water to be hauled up, in which he discovered two kinds of animals that occasioned the phenomenon; the one, a crustaceous insect which he called the cancer fulgens; the other, a large species of medusa, to which he gave the name of pellucens.

The cancer fulgens bears some resemblance to the common shrimp; it is however considerably less; the legs are furnished with numerous setæ. The light of this animal, which is very brilliant, appears to issue from every part of the body.

The medusa pellucens measures about six inches across the crown or umbella; this part is marked by a number of opake

* Hablitzl ap. Pall. n. Nord. Beytr. 4, p. 396.
† Journal de Physique, Tome XXVIII.

 lines, lines, that pass off from the center to the circumference. The edge of the umbella is divided into lobules, which succeed each other, one large and two small ones alternately. From within the margin of the umbella, there are suspended a number of long cord-shaped tentacula. The central part of the animal is opake, and furnished with four thick irregularly shaped processes, which hang down in the midst of the tentacula.

This zoophyte is the most splendid of the luminous inhabitants of the ocean. The flashes of light emitted during its contractions, are so vivid as to affect the sight of the spectator.

In the notes communicated to Sir Joseph Banks by Captain Horsburg, he remarks that the luminous state of the sea between the tropics, [illegible] generally accompanied with the appearance of a great [illegible]ber of marine animals of various kinds upon the surfa[illegible] of the water: to many of which he does not, however, attribute the property of shining. At other times, when the water which gave out light was examined, it appeared only to contain small particles of a dusky straw colour, which dissolved with the slightest touch of the finger. He likewise observes, that in Bombay, during the hot weather of May and June, he has frequently seen the edges of the sea much illuminated by minute sparkling points.

At sun-rise on April 12, 1798, in the Arabian sea, he perceived several luminous spots in the water, which conceiving to be animals, he went in the boat and caught one. It proved to be an insect somewhat resembling in appearance the wood louse, and was about one third of an inch in length. When viewed with the microscope, it seemed to be formed by sections of a thin crustaceous substance. During the time that any fluid remained in the animal, it shone brilliantly like the fire fly.

In the month of June in the same year, he picked up another luminous insect on a sandy beach, which was also covered with a thin shell, but it was a different shape, and a larger size than the animal taken in the Arabian sea.

By comparing the above description with an elegant pen and ink drawing which was made by Captain Horsburg, and accompanied his paper, I have no doubt that both these insects were monoculi; the first evidently belongs to the genus limulus of Muller; I shall therefore beg leave to distinguish it by the name of limulus noctilucus.

My pursuits, and the state of my health, having frequently led me to the coast, I have had many opportunities of making observations upon the animals which illuminate our own own seas. Of these I have discovered three species: one of which is a beroe not hitherto described by authors; another agrees so nearly with the medusa hemispherica, that I conceive it to be the same, or at least a variety of that species; the third is a minute species of medusa, which I believe to be the luminous animal, so frequently seen by navigators, although it has never been distinctly examined or scribed.

I first met with these animals in the month of October 1804, at Herne Bay, a small watering place upon the northern coast of Kent. Having observed the sea to be extremely luminous for several nights, I had a considerable quantity of the water taken up. When perfectly at rest, no light was emitted, but on the slightest agitation of the vessel in which the water was contained, a brilliant scintillation was perceived, particularly towards the surface; and when the vessel was suddenly struck, a flash of light issued from the top of the water, in consequence of so many points shining at the same moment. When any of these sparkling points were removed from the water, they no longer yielded any light. They were so transparent, that in the air they appeared like globules of water. They were more minute than the head of the smallest pin. Upon the slightest touch they broke and vanished from the sight. Having strained a quantity of the luminous water, a great number of these transparent corpuscles were obtained upon the cloth, and the water which had been strained did not afterwards exhibit the least light. I then put some sea water that had been rendered particularly clear, by repeated filtrations, into a large glass, and having floated in it a fine cloth, on which I had previously collected a number of luminous points, several of them were liberated, and became distinctly visible in their natural element, by placing the glass before a piece of dark coloured paper. They were observed to have a tendency to come to the surface of the water, and after the glass was set by for some time, they were found congregated together, and when thus collected in a body, they had a dusky straw colour, although individually they were so transparent as to be perfectly invisible, except under particular circumstances. Their substance was indeed so extremely tender and delicate, that they did not become opaque in distilled vinegar or alcohol, until immersed in these liquors for a considerable time.

On examining these minute globules with the microscope, I found that they were not quite perfect spheres, but had an irregular depression on one side, which was formed of an opaque substance, that projected a little way inwards, producing ducing such an appearance as would arise from tying the neck of a round bag, and turning it into the body.

The motions of these creatures in the water were slow and graceful, and not accompanied by any visible contraction of their bodies. After death they always subsided to the bottom of the vessel.

From the sparkling light afforded by this species, I shall distinguish it by the name of medusa scintillans.

The night following that, on which I discovered the preceding animal, I caught the two other luminous species. One of these I shall call the beroe fulgens.

This most elegant creature is of a colour changing between purple, violet, and pale blue; the body is truncated before, and pointed behind; but the form is difficult to assign, as it is varied by partial contractions, at the animal's pleasure. I have represented the two extremes of form that I have seen this creature assume: the first is somewhat that of a cucumber, which as being the one it takes when at rest, should perhaps be considered as its proper shape: the other resembles a pear, and is the figure it has in the most contracted state. The body is hollow, or forms internally an infundibular cavity, which has a wide opening before, and appears also to have a small aperture, posteriorly through which it discharges its excrement. The posterior two-thirds of the body are ornamented with eight longitudinal ciliated ribs, the processes of which are kept in such a rapid rotatory motion, while the animal is swimming, that they appear like the continual passage of a fluid along the ribs. The ciliated ribs have been described by Professor Mitchell, as arteries, in a luminous beroe, which I suspect was no other than the species I am now giving an account of.

When the beroe fulgens swam gently near the surface of the water, its whole body became occasionally illuminated in a slight degree; during its contractions, a stronger light issued from the ribs, and when a sudden shock was communicated to the water, in which several of these animals were placed, a vivid flash was thrown out. If the body were broken, the fragments continued luminous for some seconds, and being rubbed on the hand, left a light like that of phosphorus: this however, as well as every other mode of emitting light, ceased after the death of the animal.

The hemispherical species that I discovered, had a very faint purple colour. The largest that I found measured about three quarters of an inch in diameter. The margin of the umbella was undivided, and surrounded internally by a row of pale brown spots, and numerous small twisted tentacula: four opaque lines crossed in an arched manner from the the circumference, towards the center of the animal; an opaque irregular shaped process hung down from the middle of the umbella: when this part was examined with a lens of high powers, I discovered that it was inclosed in a sheath in which it moved, and that the extremity of the process was divided into four tentacula, covered with little cups or suckers, like those on the tentacula of the cuttle-fish.

This species of medusa bears a striking resemblance to the figures of the medusa hemispherica, published by Gronovius and Muller; indeed it differs as little from these figures, as they do from each other. Its luminous property, however, was not observed by these naturalists, which is the more extraordinary, as Muller examined it at night, and says it is so transparent, that it can only be seen with the light of a lamp. If it should be still considered as a distinct species, or as a variety of the hemispherica, I would propose to call it the medusa lucida.

In this species, the central part, and the spot round the margin, are commonly seen to shine on lifting the animal out of the water into the air, presenting the appearance of an illuminated wheel, and when it is exposed to the usual percussion of the water, the transparent parts of its body are alone luminous.

In the month of September, 1805, I again visited Herne Bay, and frequently had opportunities of witnessing the luminous appearance of the sea. I caught many of the hemispherical and minute species of medusa, but not one of the beroe fulgens. I observed that these luminous animals always retreated from the surface of the water, as soon as the moon rose. I found also, that exposure to the day-light took away their property of shining, which was revived by placing them for some time in a dark situation.

In that season I had two opportunities of seeing an extended illumination of the sea, produced by the above animals. The first night I saw this singular phenomenon, was extremely dark; many of the medusa scintillans, and medusa hemispherica had been observed at low water, but on the return of the tide, they had suddenly disappeared. On looking towards the sea, I was astonished to perceive a flash of light of about six yards broad extend from the shore, for apparently the distance of a mile and a half along the surface of the water. The second time that I saw this sort of light proceed from the sea, it did not take the same form, but was diffused over the surface of the waves next the shore, and was so strong, that I could for the moment distinctly see my servant, who stood at a little distance from me: he also perceived it, and called out to me at the same instant. On both these these occasions the flash was visible for about four or five seconds, and although I watched for a considerable time, I did not see it repeated.

A diffused luminous appearance of the sea, in some respects different from what I have seen, has been described by several navigators.

Godeheu de Riville saw the sea assume the appearance of a plain of snow on the coast of Malabar.*

Captain Horsburgh, in the notes he gave to Sir Joseph Banks, says, there is a peculiar phenomenon sometimes seen within a few degrees distance of the coast of Malabar, during the rainy monsoon, which he had an opportunity of observing. At midnight the weather was cloudy, and the sea was particularly dark, when suddenly it changed to a white flaming colour all around. This bore no resemblance to the sparkling or glowing appearance he had observed on other occasions in seas near the equator, but was a regular white colour, like milk, and did not continue more than ten minutes. A similar phenomenon, he says, is frequently seen in the Banda sea, and is very alarming to those who have never perceived or heard of such an appearance before.

This singular phenomenon appears to be explained by some observations communicated to me by Mr. Langstaff, a surgeon in the city, who formerly made several voyages. In going from New Holland to China, about half an hour after sunset, every person on board was astonished by a milky appearance of the sea: the ship seemed to be surrounded by ice covered with snow. Some of the company supposed they were in soundings, and that coral bottom gave this curious reflection, but on sounding with 70 fathoms of line no bottom was met with. A bucket of water being hauled up, Mr. Langstaff examined it in the dark, and discovered a great number of globular bodies, each about the size of a pin's head, linked together. The chains thus formed did not exceed three inches in length, and emitted a pale phosphoric light. By introducing his hand into the water, Mr. Langstaff raised upon it several chains of the luminous globules, which were separated by opening the fingers, but readily re-united on being brought again into contact, like globules of quicksilver. The globules, he says, were so transparent, that they could not be perceived when the hand was taken into the light.

This extraordinary appearance of the sea was visible for

* Mem. Etrang. de l'Acad. des Sc. Tom. 3.

two two nights. As soon as the moon exerted her influence, the sea changed to its natural dark colour, and exhibited distinct glittering points, as at other times. The phenomenon, he says, had never been witnessed before by any of the company on board, although some of the crew had been two or three times round the globe.

I consider this account of Mr. Langstaff very interesting and important, as it proves that the diffused light of the sea is produced by an assemblage of minute medusæ on the surface of the water.

In June, 1806, I found the sea at Margate more richly stored with the small luminous medusæ, than I have ever seen it. A bucket of the water being set by for some time, the animals sought the surface, and kept up a continual sparkling, which must have been occasioned by the motions of individuals, as the water was perfectly at rest. A small quantity of the luminous water was put into a glass jar, and on standing some time, the medusæ collected at the top of the jar, and formed a gelatinous mass, one inch and a half thick, and of a reddish or mud colour, leaving the water underneath perfectly clear.

In order to ascertain if these animals would materially alter their size, or assume the figure of any other known species of medusa, I kept them alive for twenty-five days, by carefully changing the water in which they were placed; during which time, although they appeared as vigorous as when first taken, their form was not in the slightest degree altered, and their size but little increased. By this experiment I was confirmed in the opinion of their being a distinct species, as the young actinæ and medusæ exhibit the form of the parent in a much shorter period than the above.

In September 1806, I took at Sandgate a number of the beroe fulgens, but no other species: they were of various dimensions, from the full size down to that of the medusa scintillans: they could however be clearly distinguished from the latter species, by their figure.

Since that time, I have frequently met with the medusa scintillans on different parts of the coast of Sussex, at Tenby, and at Milford-haven. I have likewise seen this species in the bays of Dublin and Carlingford in Ireland.

In the month of April, last year, I caught a number of the beroe fulgens in the sea at Hastings; they were of various sizes, from about the half of an inch in length, to the bulk of the head of a large pin. I found many of them adhering together in the sea; some of the larger sort were covered with small ones, which fell off when the animals were handled, and by a person unaccustomed to observe these creatures, would have been taken for a phosphoric substance. On putting a number of them into a glass, containing clear sea water, they still shewed a disposition to congregate upon the surface. I observed that when they adhered together, they shewed no contractile motion in any part of their body, which explains the cause of the pale or white colour of the diffused light of the ocean. The flashes of light which I saw come from the sea at Herne bay, were probably produced by a sudden and general effort of the medusæ to separate from each other, and descend in the water.

The medusa scintillans almost constantly exists in the different branches of Milford-haven that are called pills. I have sometimes found these animals collected in such vast numbers in those situations, that they bore a considerable proportion to the volume of the water in which they were contained: thus, from a gallon of sea water in a luminous state, I have strained above a pint of these medusæ. I have found the sea under such circumstances to yield me more support in swimming, and the water to taste more disagreeably than usual; probably the difference of density, that has been remarked at different times in the water of the sea, may be referred to this cause.

All my own observations lead me to conclude, that the medusa scintillans is the most frequent source of the light of the sea around this country, and by comparing the accounts of others with each other, and with what I have myself seen, I am persuaded that it is so likewise in other parts of the world. Many observers appear to have mistaken this species for the nereis noctiluca, which was very natural, as they were prepossessed with the idea of the frequent existence of the one, and had no knowledge of the other. Some navigators have actually described this species of medusa, without being aware of its nature. Mr. Bajon, during his voyage from France to Cayenne, collected many luminous points in the sea, which, he says, when examined by a lens, were found to be minute spheres. They disappeared in the air. Doctor Le Roy, in sailing from Naples to France, observed the sparkling appearance of the sea, which is usually produced by the medusa scintillans. By filtering the water, he separated luminous particles from it, which he preserved in spirits of wine: they were, he says, like the head of a pin, and did not at all resemble the nereis noctiluca, described by Vianelli; their colour approached a yellow brown, and their substance was extremely tender, and fragile. Notwithstanding this striking resemblance to the medusa scintillans, Le Roy, in consequence of a preconceived theory, did not suppose pose what he saw were animals, but particles of an oily or bituminous nature.*

The minute globules seen by Mr. Langstaff in the Indian ocean were, I think, in all probability, the scintillating species of medusa; and on my shewing him some of these animals I have preserved in spirits, he entertained the same opinion.

Professor Mitchell, of New York, found the luminous appearance on the coast of America to be occasioned by minute animals, that from his description, plainly belonged to this species of medusa, notwithstanding which, he supposed them to be a number of the nereis noctiluca.†

The luminous animalcule, discovered by Forster off the Cape of Good Hope, in his voyage round the world, bears so strong a resemblance to the medusa scintillans, that I am much disposed to believe them the same. He describes his animalcule as being a little gelatinous globule, less than the head of a pin; transparent, but a little brownish in its colour; and of so soft a texture, that it was destroyed by the slightest touch. On being highly magnified, he perceived on one side a depression, in which there was a tube that passed into the body, and communicated with four or five intestinal sacs. The pencil drawings he made on the spot are in the possession of Sir Joseph Banks. By comparing these with the representations of the medusa scintillans, and some of this species rendered visible, by being a long time preserved in spirits, which I have laid before this learned Society, it will be found, that the only difference between Forster's animalcule, and the medusa scintillans, is in the appearance of the opaque parts, shewn by the microscope.

Many writers have ascribed the light of the sea to other causes than luminous animals. Martin supposed it to be occasioned by putrefaction: Silberschlag believed it to be phosphoric: Professor J. Mayer conjectured, that the surface of the sea imbibed light, which it afterwards discharged. Bajon and Gentil thought the light of the sea was electric, because it was excited by friction. Forster conceived that it was sometimes electric, sometimes caused from putrefaction, and at others by the presence of living animals. Fougeroux de Bondaroy believed that it came sometimes from electric fires, but more frequently from the putrefaction of marine animals and plants.

---

* Observ. sur un lumiere produite par L'Eau de la mer. Mem. Etrang. des Sc.

† Phil. Mag. Vol. X. p. 20.

I shall not trespass on the time of the Society, to refute the above speculations; their authors have left them unsupported by either arguments or experiments, and they are inconsistent with all ascertained facts upon this subject.

The remarkable property of emitting light during life, is only met with amongst animals of the four last classes of modern naturalists, viz. MOLLUSCA, INSECTS, WORMS, and ZOOPHYTES.

The mollusca and worms contain each but a single luminous species; the *pholas dactylus* in the one, and the *nereis noctiluca* in the other.

Some species yield light, in the eight following genera of insects; *elater*, *lampyris*, *fulgora*, *pausus*, *scolopendra*, *cancer*, *lynceus*,* and *limulus*. The luminous species of the genera lampyris, and fulgora, are more numerous than is generally supposed, if we may judge from the appearance of luminous organs to be seen in dried specimens.

Amongst zoophytes we find that the genera *medusa*, *beroe*,† and *pennatula*, contain species which afford light.

The only animals which appear to possess a distinct organization for the production of light, are the luminous species of lampyris, elater, fulgora, and pausus.

The light of the lampyrides is known to proceed from some of the last rings of the abdomen, which when not illuminated, are of a pale yellow colour. Upon the internal surface of these rings, there is spread a layer of a peculiar soft yellow substance, which has been compared to paste, but by examination with a lens, I found it to be organized like the common interstitial substance of the insect's body, except that it is of a closer texture, and a paler yellow colour. This substance does not entirely cover the inner surface of the rings, being more or less deficient along their edges, where it presents an irregular waving outline. I have observed in the glow worm, that it is absorbed, and its place supplied by common interstitial substance, after the season for giving light is past.

The segments of the abdomen, behind which this peculiar substance is situated, are thin and transparent, in order to expose the internal illumination.

---

* The animal discovered by Riville off the coast of Malabar in 1754, is certainly a testaceous insect, and appears to belong to the genus lynceus of Muller.

† The luminous zoophyte for which Peron has lately instituted the new genus pyrosoma, appears to me to be a beroe, and only worthy of a specific distinction.

The

The number of luminous rings varies in different species of lampyris, and as it would seem at different periods in the same individual.

Besides the luminous substance above described, I have discovered in the common glow worm, on the inner side of the last abdominal ring, two bodies, which to the naked eye appear more minute than the head of the smallest pin. They are lodged in two slight depressions, formed in the shell of the ring, which is at these points particularly transparent. On examining these bodies under the microscope, I found that they were sacs containing a soft yellow substance, of a more close and homogeneous texture, than that which lines the inner surface of the rings. The membrane forming the sacs, appeared to be of two layers, each of which is composed by a transparent silvery fibre, in the same manner as the internal membrane of the respiratory tubes of insects, except that in this case, the fibre passes in a spiral, instead of a circular direction. This membrane, although so delicately constructed, is so elastic as to preserve its form, after the sac is ruptured, and the contents discharged.

The light that proceeds from these sacs, is less under the controul of the insect, than that of the luminous substance spread on the rings: it is rarely ever entirely extinguished in the season that the glow worm gives light, even during the day; and when all the other rings are dark, these sacs often shine brightly.

The circumstance of there being points, which give a more permanent light than the other parts of the luminous rings of the abdomen, has been noticed before by the Comte G. de Razoumouski. He states the number of these luminous points to vary from 2 to 5.*

I must however remark, that I never saw more than two of these luminous points, which were always upon the last ring of the body, and that the figures which accompany the memoir of the Comte de Razoumousky, bear scarcely any resemblance to the insect they are intended to represent, from which we may fairly suspect him of inaccuracy in other particulars.

As far as my observation has extended, the small sacs of luminous substances are not found in any species of lampyris, except the glow worm of this country. Thunburg mentions that the lampyris japonica has two vesicles on the tail, which afford light.

The organs for the production of light in the genus elater, are situated in the corcelet; these likewise consist of a pecu-

* Mem. de la Soc. de Lausanne, Tom. ii.

liar liar yellow substance, placed behind transparent parts of the shell, which suffer the natural colour of this substance to be seen through them in the day, and when illuminated, give passage to the light.

On dissecting the organs of light in the elater noctilucus, I found that there is a soft yellow substance, of an oval figure, lodged in the concavity of the yellow spots of the corcelet, which parts are particularly thin and transparent in this species. This substance is so remarkably close in its structure, that at first view it appears like an inorganic mass, but with a lens, it is readily perceived to be composed of a great number of very minute parts or lobules closely pressed together. Around these oval masses, the interstitial substance of the corcelet is arranged in a radiated manner, and the portion of the shell that immediately covers the irradiated substance, is in a certain degree transparent, but less so than that which lies over the oval masses; it is therefore probable, that the interstitial substance in this situation may be endowed with the property of shining. A fasciculus of the muscles of the corcelet arises in the interior of the oval masses of the luminous substance, but not apparently with any design, as it contributes, with the adjacent fasciculi, to move the anterior feet.

In the elater ignitus, the masses of luminous substance are extremely irregular in their figure: they are situated nearly at the posterior angles of the corcelet, and are more loose in their texture than the oval masses of the noctilucus, resembling rather in composition, the interstitial substance which surrounds these masses in that species. The shell of the corcelet is somewhat thinner, and more transparent along both sides of the margin, than at other places, but it is not, as in the noctilucus, elevated, and peculiarly clear and thin immediately, over the seat of the luminous organ; consequently, the light emitted by the elater ignitus cannot be very brilliant.

I have not been able to procure any specimen of the elater phosphorea, but from the accounts of naturalists, it appears to resemble in every respect the elater noctilucus; indeed I have great doubts of the phosphorea being a distinct species.

I have had an opportunity of examining, preserved in a moist way, two species of fulgora, the candelaria and lanternaria. The light in this genus has been observed to issue from the remarkable proboscis on the fore part of the head. This part has always been described by authors as hollow or empty, which I have found to be perfectly correct; and what is more extraordinary, that the cavity communicates freely with with the external air, by means of a chink or narrow aperture, placed on each side of the root of the proboscis. This projection is covered internally by a membrane, between which and the horny part or shell, there appears to be interposed a pale reddish coloured soft substance, that is arranged in the candelaria in broad lines or stripes; but it is so thin, that I could not distinctly examine its structure, or absolutely determine, whether it should be considered as a substance intended to furnish the light of these insects, or the pigment upon which the colour of the proboscis depends.

The globes of the antennæ constitute the organs of light in the pausus spherocerus. Dr. Afzelius, who discovered the luminous property in this species, compares them to lanterns spreading a dim phosphoric light.* The rarity of the insect put it out of my power to examine its structure, but from the form and situation of its organs of light, it is most probable they are constructed like those of the fulgoræ.

It has been conjectured by Carradori and others, that the lampyrides were enabled to moderate or extinguish their light, by retracting the luminous substance under a membrane; but neither in them, or any of the other luminous insects, have I found an apparatus of this sort. The substance furnishing the light, is uniformly applied to corresponding transparent parts of the shell of the insect from whence it is not moved; indeed a membrane, if it did exist, would have but little effect in obscuring the light, and never could serve to extinguish it. The regulation of the kind and degree of the luminous appearance does not depend upon any visible mechanism, but like the production of the light itself, is accomplished by some inscrutable change in the luminous matter, which in some animals is a simple operation of organic life, and in others is subject to the will.

It is worthy of remark, that in all the dissections I have made of luminous insects, I did not find that the organs of light were better, or differently supplied with either nerves or air tubes, than the other parts of the body. The power of emitting light likewise exists in many creatures which want nerves, a circumstance strongly marking a difference between animal light, and animal electricity.

With the exception of the animals above mentioned, the exhibition of light depends upon the presence of a fluid matter.

In the pholas dactylus, the luminous fluid is particularly evident, and in vast quantity; it is recorded by Pliny, that this fluid is like liquid phosphorus, and renders every object

* Lin. Trans. Vol. IV.

luminous luminous with which it comes into contact. Reaumur also found that it was diffusible in water, or any other fluid in which the animal might be immersed.*

The shining of the scolopendra electrica, I have always observed to be accompanied by the appearance of an effusion of a luminous fluid upon the surface of the animal, more particularly about the head, which may be received upon the hand, or other bodies brought into contact with the insect at the moment, and these exhibit a phosphoric light for a few seconds afterwards. This fluid, however, I never could discover in the form of moisture, even upon the clearest glass, although examined immediately with the most scrupulous attention by a lens: it must therefore be extremely attenuated.

The same appearance has been observed during the illumination of the nereis noctiluca by Fougeroux de Bondaroy.†

The animal discovered by Riville shed a blue liquor, which illuminated the water for a distance of two or three lines.‡

Spallanzani relates, that the medusa which he examined communicated the property of shining to water, milk, and other fluids, on being rubbed or squeezed in them. §

The luminous fluid is in some instances confined to particular parts of the body, and in others is diffused throughout the whole substance of the animal.

In the scolopendra electrica, it appears to reside immediately under the integuments. In the lynceus discovered by Riville, it is contained in the ovary. If I may judge from my own observations, every part of the body of the medusæ is furnished with this fluid, as there is no part I have not seen illuminated under different circumstances; but Spallanzani affirms that it is only found in the large tentacula, the edges of the umbella, and the purse or central mass; which he proved, he says, by detaching these parts successively, when they shone vividly, while the rest of the body neither gave light or communicated any luminous appearance to water.||

Spallanzani discovered a mucous luminous fluid in the plumule of the pennatula phosphorea.¶

(*To be continued.*)

---

* Mem. de l'Acad. des Sc. 1712.

† Mem. de l'Acad. des Sc. 1767.

‡ Mem. Etrang de l'Acad. des Sc. Tom. iii.

§ Spallanzani's Travels in the Two Sicilies, Vol. iv.

|| Memoria sopra le meduse fosforiche. Mem. della Soc. Ital. Tomo vii.

¶ Mem. della Soc. Ital. Tomo ii.

